# Estimating linkage disequilibrium from genotypes under Hardy-Weinberg equilibrium

**DOI:** 10.1186/s12863-020-0818-9

**Published:** 2020-02-26

**Authors:** Tin-Yu J. Hui, Austin Burt

**Affiliations:** Department of Life Sciences, Silwood Park Campus, Imperial College London, Ascot, Berkshire SL5 7PY UK

**Keywords:** Linkage disequilibrium, Maximum likelihood estimation, Sampling error

## Abstract

**Background:**

Measures of linkage disequilibrium (LD) play a key role in a wide range of applications from disease association to demographic history estimation. The true population LD cannot be measured directly and instead can only be inferred from genetic samples, which are unavoidably subject to measurement error. Previous studies of *r*^2^ (a measure of LD), such as the bias due to finite sample size and its variance, were based on the special case that the true population-wise LD is zero. These results generally do not hold for non-zero ${r}_{true}^2$ values, which are more common in real genetic data.

**Results:**

This work generalises the estimation of *r*^2^ to all levels of LD, and for both phased and unphased data. First, we provide new formulae for the effect of finite sample size on the observed *r*^2^ values. Second, we find a new empirical formula for the variance of the observed *r*^2^, equals to 2*E*[*r*^2^](1 − *E*[*r*^2^])/*n*, where *n* is the diploid sample size. Third, we propose a new routine, Constrained ML, a likelihood-based method to directly estimate haplotype frequencies and *r*^2^ from diploid genotypes under Hardy-Weinberg Equilibrium. While serving the same purpose as the pre-existing Expectation-Maximisation algorithm, the new routine can have better convergence and is simpler to use. A new likelihood-ratio test is also introduced to test for the absence of a particular haplotype. Extensive simulations are run to support these findings.

**Conclusion:**

Most inferences on LD will benefit from our new findings, from point and interval estimation to hypothesis testing. Genetic analyses utilising *r*^2^ information will become more accurate as a result.

## Background

### Introduction

Linkage Disequilibrium (LD) was first defined about 100 years ago as the non-random association of alleles at different loci [1]. Since that time there has been much research on the topic, some focused on how LD is quantified and defined [2–7], and a larger fraction on the connection between LD and various evolutionary forces that shape it, including genetic drift [8–11] and selection [12, 13]. These investigations have also extended to sub-divided or structured populations [14–17]. In principle, these theoretical works allow one to infer features of the underlying processes from measures of LD [18, 19]. Another application of LD includes association studies to identify genes for diseases, such as in the Human Haplotype Map project [20]. With the advance in sequencing technology, computer packages have been developed to calculate LD for large numbers of samples and genetic loci [5, 21–24]. While there are plenty of applications utilising LD information, they all rely on accurate and robust estimation of the parameter of interest, which many have taken for granted. There are, however, key gaps regarding LD estimation that have yet to be resolved.

The squared correlation coefficient *r*^2^ is a popular measure of LD alongside *D* or *D*’ [1]. One advantage of *r*^2^ is that it is less sensitive to marginal allele frequencies. It also relates to the *ϕ* correlation coefficient and *χ*^2^ test statistic for association of contingency tables [25]. Further, Sved and Feldman [26] showed the equivalence of *r*^2^ and the probability of linked identity by decent between two random-chosen haplotypes. Most previous studies concerning the estimation of *r*^2^, including the mean and variance, have been based on the assumption of linkage equilibrium (i.e. ${r}_{true}^2=0$). These findings do not hold for real datasets where the true correlation between loci is non-zero. In this article we extend the theory of *r*^2^ estimation to all levels of LD. We first study the expectation of the observed *r*^2^ for finite sample size, as sampling is known to bias the observed *r*^2^ [27]. Second, we approximate the empirical variance of the observed *r*^2^ as a function of sample size and its expectation *E*[*r*^2^]. Third, we propose a direct routine to estimate haplotype frequencies and *r*^2^ for unphased data under Hardy-Weinberg Equilibrium (HWE). Throughout this paper, we define ${r}_{true}^2$ as the true population-wise, unobserved LD between two loci, while ${r}_{phased}^2$ and ${r}_{unphased}^2$ as the raw squared coefficient computed directly from phased and unphased data respectively.

### Effect of finite sample size

Consider a classical two-allele, two-locus scenario, with alleles *A* and *a* at the first locus and alleles *B* and *b* at the second. Let *p*_*AB*_, *p*_*Ab*_, *p*_*aB*_, *p*_*ab*_ be the true haplotype frequencies of the four haplotype combinations *AB*, *Ab*, *aB*, *ab*. Statistically speaking, if samples are taken with replacement, the observed haplotype counts follow a multinomial distribution with size 2*n* and probabilities equal the true haplotype frequencies. Let $\overset{\sim }{p_{AB}},\overset{\sim }{p_{Ab}},\overset{\sim }{p_{aB}},\overset{\sim }{p_{ab}}$ be the sampled haplotype frequencies from our genetic samples, which are also the maximum likelihood estimators (MLE) for the true haplotype frequencies. We also let ${r}_{phased}^2$ be the squared correlation computed directly using the observed frequencies:

1 $$ {r}_{phased}^2=\frac{{\left(\overset{\sim }{p_{AB}}\overset{\sim }{p_{ab}}-\overset{\sim }{p_{Ab}}\overset{\sim }{p_{aB}}\right)}^2}{\overset{\sim }{p_A}\ \left(1-\overset{\sim }{p_A}\right)\overset{\sim }{p_B}\left(1-\overset{\sim }{p_B}\ \right)} $$

where $\overset{\sim }{p_A}=\overset{\sim }{p_{AB}}+\overset{\sim }{p_{Ab}}$ and $\overset{\sim }{p_B}=\overset{\sim }{p_{AB}}+\overset{\sim }{p_{aB}}$ are the observed marginal allele frequencies for allele *A* and *B*. Note that this formula is identical to the square of the *ϕ* coefficient for a two-by-two contingency table [25, 28]. The invariant principle of MLE suggests that ${r}_{phased}^2$ is also the MLE for ${r}_{true}^2$, but does not guarantee its unbiasedness towards the parameter of interest. The next step is to establish the effect of sample size and find a formula to connect ${r}_{phased}^2$ and ${r}_{true}^2$.

Sved and Feldman [26] showed the expected change in *r*^2^ due to genetic drift over two successive generations is

2 $$ E\left[{r}_{t+1}^2\right]=\frac{1}{2{N}_e}+\left(1-\frac{1}{2{N}_e}\right){\left(1-c\right)}^2{r}_t^2 $$

with *c* being the recombination rate between a pair of loci and *N*_*e*_ the effective population size. This equation is seemingly irrelevant to our problem, but we may consider the sampling process as another generation of genetic drift with population size equal to the sample size 2*n* under complete linkage (*c* = 0). Therefore, given the true ${r}_{true}^2$ for a population, the expected observed ${r}_{phased}^2$ becomes:

3 $$ E\left[{r}_{phased}^2\right]=\frac{1}{2n}+\left(1-\frac{1}{2n}\right){r}_{true}^2 $$

Or when we estimate the underlying ${r}_{true}^2$ from an observed value ${r}_{phased}^2$ the sample size correction formula becomes:

4 $$ \hat{r_{true}^2}=\frac{r_{phased}^2-\frac{1}{2n}}{1-\frac{1}{2n}} $$

For unphased data, the sample size correction should largely follow the phased case, with *n* replacing 2*n*:

5 $$ E\left[{r}_{unphased}^2\right]=\frac{1}{n}+\left(1-\frac{1}{n}\right){r}_{true}^2 $$

and similarly if we estimate the underlying ${r}_{true}^2$ from the estimated haplotype frequencies, the sample size correction formula is:

6 $$ \hat{r_{true}^2}=\left({r}_{unphased}^2-\frac{1}{n}\right)/\left(1-\frac{1}{n}\right) $$

### Empirical variance of *r*^2^

*r*^2^ is a ratio hence its variance is difficult to evaluate. The variance is required when inferring the confidence interval (C.I.) of an *r*^2^ estimate from a pair of loci, or in hypothesis testing to test against a specific true value. Many existing applications, such as those for effective population size estimation, suggest that the observed $\frac{r^2}{E\left[{r}^2\right]}$ is approximately *χ*^2^ distributed with 1 degree of freedom, which implies that *var*(*r*^2^) ≈ 2*E*[*r*^2^]^2^ [27, 29]. This expression is derived under the null distribution of the *χ*^2^ statistic and is only correct if the underlying ${r}_{true}^2=0$. An obvious counter-example is a pair of perfectly correlated loci, whose ${r}_{true}^2$ and observed ${r}_{phased}^2$ (or ${r}_{unphased}^2$) are 1, and hence the variance is 0 (instead of 2). While a closed-form expression for the variance may not exist, we will approximate it with empirical simulations and relate it to sample sizes and other factors.

### Estimating haplotype frequencies from unphased data

The term LD is often called the “gametic phase disequilibrium”, which specifically refers the correlation of alleles at the haplotype level. For diploid individuals, however, direct inference of haplotype frequencies is usually impossible when gametic phase is not known. The reason is that we are unable to tell the exact haplotype configuration for double heterozygotes, as they can be *AB*/*ab* or *Ab*/*aB*. Under HWE the expected frequencies for each genotype *f*_1_, *f*_2_, …, *f*_9_ are shown in Table 1. As introduced by Hill in 1974, the log-likelihood with respect to the haplotype frequencies, is [2]:

7 $$ l\left({p}_{AB},{p}_{Ab},{p}_{aB},{p}_{ab}\right)= constant+\sum \limits_{i=1}^9{n}_i\log \left({f}_i\right) $$

where *n*_1_, *n*_2_, …, *n*_9_ are the counts for each genotype. It is easy to understate the challenges in maximising this log-likelihood. Direct maximisation of Eq. 7 is not always feasible, hence the use of Expectation-Maximisation (EM) algorithm was suggested [21]. The second approach, adapted by CubeX, calculates the first derivataes of Eq. 7 and solves the associated cubic equation. This however works only for the two-allele two-locus case.

**Table 1:** Expected genotypic frequencies under HWE

	$BB$	$Bb$	$bb$
$AA$	${f}_1={p}_{AB}^2$	${f}_2=2{p}_{AB}{p}_{Ab}$	${f}_3={p}_{Ab}^2$
$Aa$	${f}_4=2{p}_{AB}{p}_{aB}$	${f}_5=2\left({p}_{AB}{p}_{ab}+{p}_{Ab}{p}_{aB}\right)$	${f}_6=2{p}_{Ab}{p}_{ab}$
$aa$	${f}_7={p}_{aB}^2$	${f}_8=2{p}_{aB}{p}_{ab}$	${f}_9={p}_{ab}^2$

The expected frequency of genotypes given the haplotype frequencies under HWE [2]. All the expected frequencies ${f}_1,{f}_2,\dots, {f}_9$ add up to one

Here we propose a new approach to directly maximise Eq. 7 and thus to estimate the haplotype frequencies. Without loss of generality we drop the term *p*_*ab*_ as the four haplotype frequencies must add to one. The feasible region of the remaining three haplotype frequencies looks like a tetrahedron with vertices (1, 0, 0), (0, 1, 0), (0, 0, 1), and (0, 0, 0). Our method, called Constrained ML, transforms the haplotype frequencies before maximising the log-likelihood function. For this two-allele two-locus scenario, the transformation is as follows:

8 $$ {\displaystyle \begin{array}{l}u={p}_{AB}+{p}_{Ab}+{p}_{aB}\\ {}v=\frac{p_{AB}+{p}_{Ab}}{p_{AB}+{p}_{Ab}+{p}_{aB}}\\ {}w=\frac{p_{AB}}{p_{AB}+{p}_{Ab}}\end{array}} $$

The feasible region of the new coordinates {*u*, *v*, *w*} becomes a unit cube. The log-likelihood is then maximised with respect to the new coordinates in this “box-like” constraint, where a number of common optimisation routines become available. The MLE for the haplotype frequencies can be obtained by back transforming the $\left\{\hat{u},\hat{v},\hat{w}\right\}$ values which maximise the function.

Sometimes, we need to decide whether a haplotype actually exists in the population. For example, if *n*_6_ = *n*_8_ = *n*_9_ = 0 then we cannot rule out the possibility of *p*_*ab*_ = 0, even if the estimated frequency is not. The same principle applies to the other haplotypes. While CubeX provides an additional solution (denoted as the *γ* solution) should this happen, it gives little indication of which set of estimated haplotype frequency we should accept. Under this scenario, we propose to perform a likelihood-ratio test (LRT), to test whether a particular haplotype has zero frequency as a precaution. This use of an LRT will be demonstrated in the analysis of a real dataset.

## Results

**Fig. 1:**
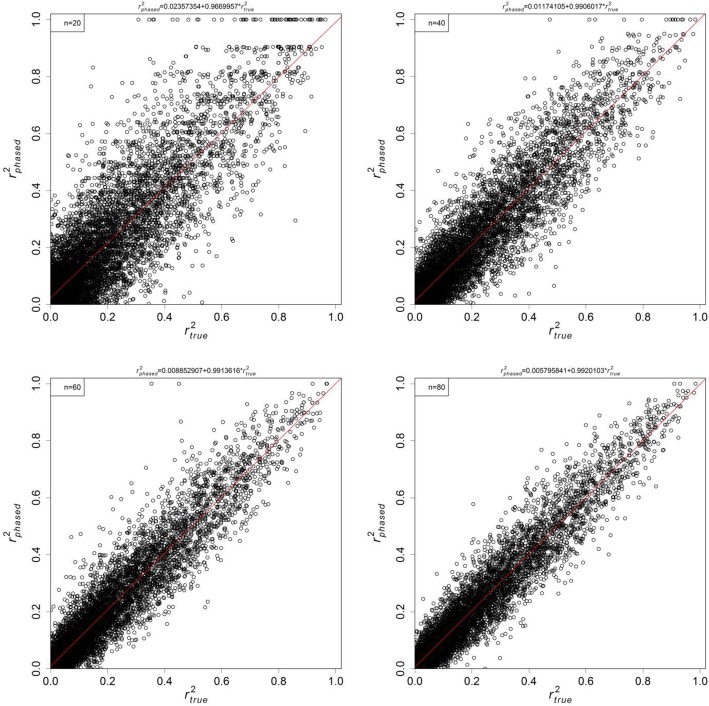
Plots of ${r}_{phased}^2$ against ${r}_{true}^2$ under different sample sizes: 20 (top left), 40 (top right), 60 (bottom left), and 80 (bottom right). A linear regression (red line) is fitted to each plot and the estimates are reported in Table 2

**Fig. 2:**
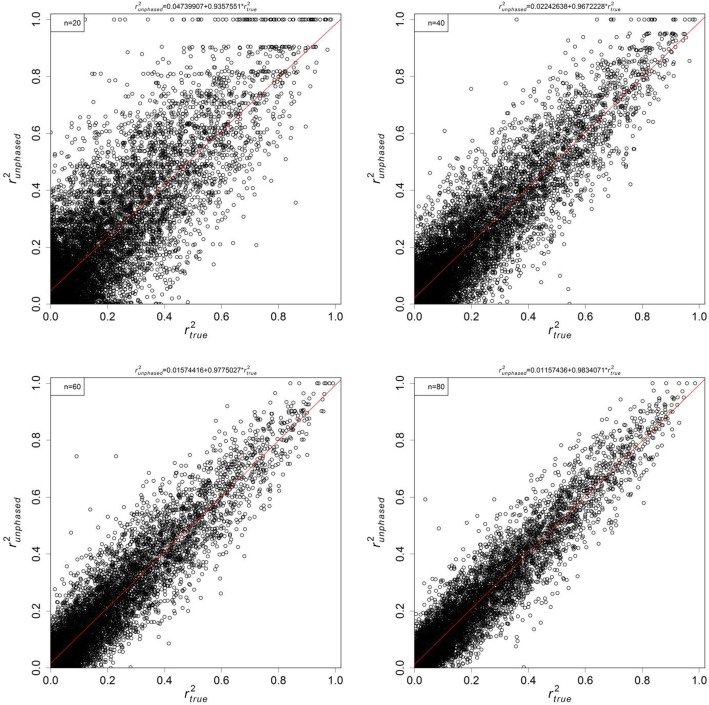
Plots of ${r}_{unphased}^2$ against ${r}_{true}^2$ under different sample sizes: 20 (top left), 40 (top right), 60 (bottom left), and 80 (bottom right). A linear regression (red line) is fitted to each plot and the estimates are reported in Table 3. Simulation setting is described in text

The plots of ${r}_{phased}^2$ versus ${r}_{true}^2$ are shown in Fig. 1 for several sample sizes. Linear regressions were run through these simulated data points, and the estimates and confidence intervals (C.I.s) of the slopes and intercepts are summarised in Table 2. The estimates of intercepts and slopes were very close to 1/2*n* and (1 − 1/2*n*), which agree to our derivation for *r*^2^ under finite sample size $E\left[{r}_{phased}^2\right]=\left(1-\frac{1}{2n}\right){r}_{true}^2+\frac{1}{2n}$ in Eq. 3. In particular, the 95% C.I. for slopes excluded 1 for all examined cases. The results from the same study using genotypic (unphased) data are shown in Fig. 2 and Table 3. The results were similar to the phased case, with estimates of intercepts and slopes of about 1/*n* and (1 − 1/*n*) respectively. In short, both phased and unphased simulations followed closely our theoretical expectations of observed *r*^2^ due to the effect of finite sampling.

**Table 2:** Slope and intercept estimates from phased data

*n*	1/(2*n*)	Intercept estimate	1 − 1/(2*n*)	Slope estimate
20	0.025	0.02357 [0.02096, 0.02619]	0.975	0.96700 [0.95638, 0.97761]
40	0.0125	0.01174 [0.00985, 0.01363]	0.9875	0.99060 [0.98293, 0.99827]
60	0.0083	0.00885 [0.00731, 0.01040]	0.9917	0.99136 [0.98506, 0.99766]
80	0.0063	0.00580 [0.00447, 0.00712]	0.9937	0.99201 [0.98665, 0.99738]

Slope and intercept estimates for the plots in Fig. 1. 95% C.I.s are reported in brackets

**Table 3:** Slope and intercept estimates from unphased data

*n*	1/*n*	Intercept estimate	1 − 1/*n*	Slope estimate
20	0.05	0.04740 [0.04451, 0.05029]	0.95	0.93576 [0.92390, 0.94761]
40	0.025	0.02243 [0.02038, 0.02447]	0.975	0.96722 [0.95907, 0.97537]
60	0.0167	0.01574 [0.01398, 0.01750]	0.9833	0.97750 [0.97029, 0.98472]
80	0.0125	0.01157 [0.01009, 0.01306]	0.9875	0.98340 [0.97741, 0.98941]

Slope and intercept estimates for the plots in Fig. 2. 95% C.I.s are reported in brackets

**Fig. 3:**
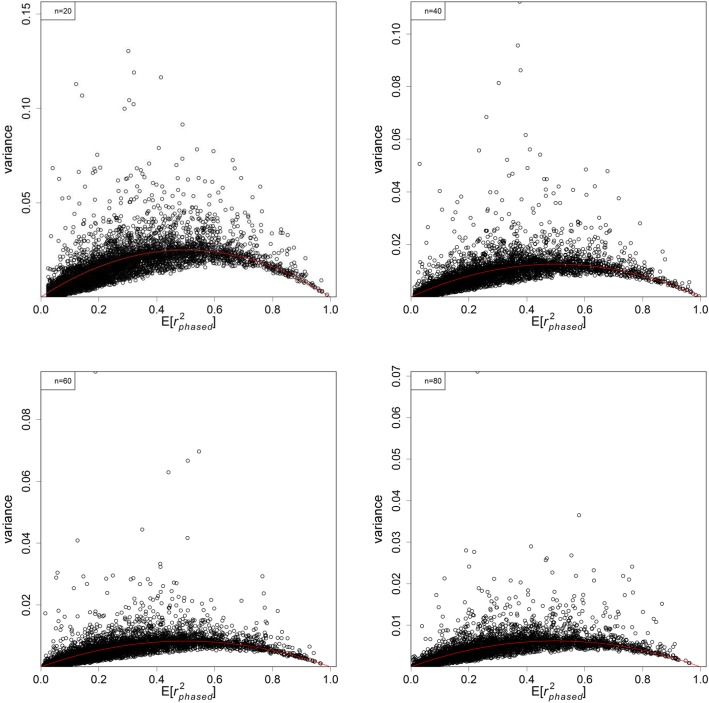
Plots of variance of ${r}_{phased}^2$ against $E\left[{r}_{phased}^2\right]$ under different sample sizes: 20 (top left), 40 (top right), 60 (bottom left), and 80 (bottom right). The red lines shows the functional form of $2E\left[{r}_{phased}^2\right]\left(1-E\left[{r}_{phased}^2\right]\right)/n$

**Fig. 4:**
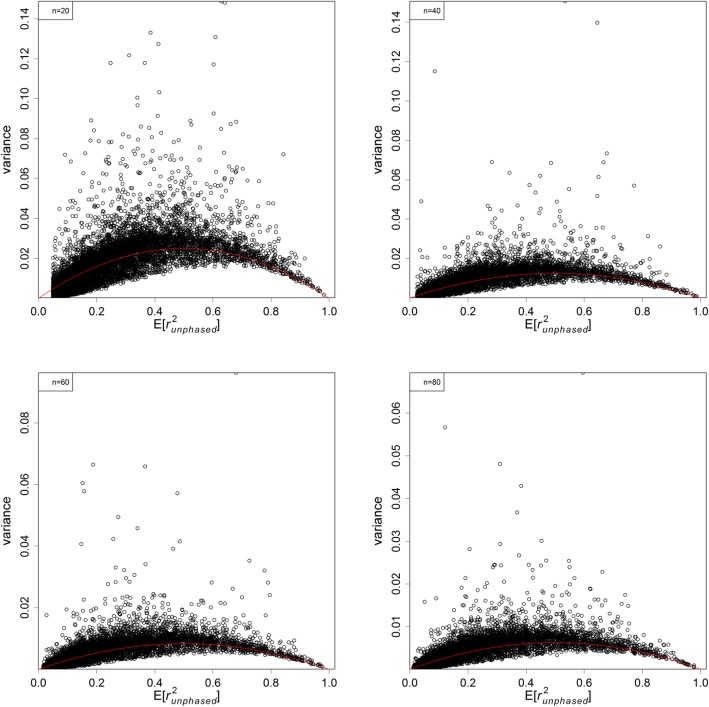
Plots of variance of ${r}_{unphased}^2$ against $E\left[{r}_{unphased}^2\right]$ under different sample sizes: 20 (top left), 40 (top right), 60 (bottom left), and 80 (bottom right). The red lines shows the functional form of $2E\left[{r}_{unphased}^2\right]\left(1-E\left[{r}_{unphased}^2\right]\right)/n$

Figures 3 and 4 show the variance plots against their expectations for phased and unphased data. The variance decreased with sample size *n*. Under the same condition the variances were smaller for phased than unphased data. The variance generally increases with their expectations for *E*[*r*^2^] < 0.5, and then come down afterwards. As predicted, the variance goes down to 0 when *E*[*r*^2^] approaches 1.

**Fig. 5:**
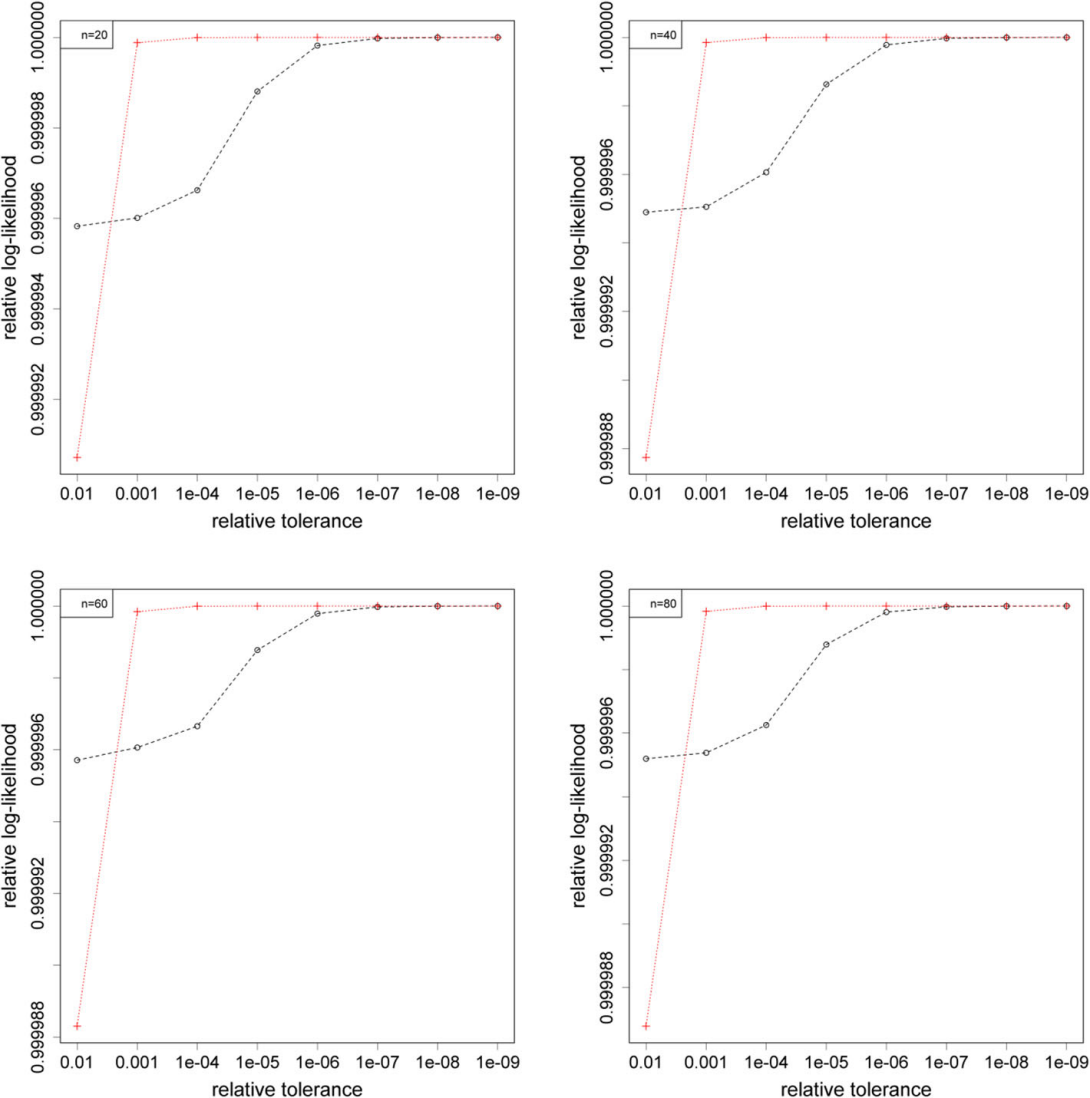
Plots of relative log-likelihood against relative tolerance for the two maximisation routines using unphased data: the EM algorithm (black circles), and Constrained ML (red crosses). Four different sample sizes were examined: 20 (top left), 40 (top right), 60 (bottom left), and 80 (bottom right). The global maximum of the log-likelihood has the relative value of 1

**Fig. 6:**
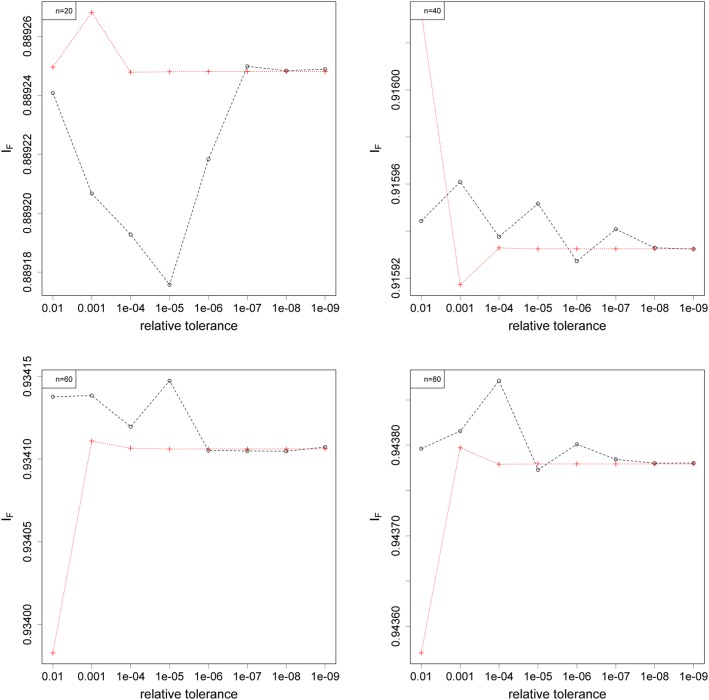
Plots of *I*_*F*_ index against relative tolerance for the two maximisation routines using unphased data: the EM algorithm (black circles), and Constrained ML (red crosses). Four different sample sizes were examined: 20 (top left), 40 (top right), 60 (bottom left), and 80 (bottom right)

Our final set of simulations compared the convergence between Constrained ML and the EM algorithm. For both methods, the maximisation terminated at the *k*^*th*^ iteration when ∣*l*^(*k* + 1)^ − *l*^(*k*)^ ∣ / max(|*l*^(*k* + 1)^|, |*l*^(*k*)^|, 1) was smaller than the chosen relative tolerance. The plots of relative log-likelihood against relative tolerance are found in Fig. 5. The global maximum of the log-likelihood surface will have the relative value of 1, and all other points will have values smaller than 1. For very loose relative tolerance of 10^−2^ Constrained ML was inaccurate. Between 10^−3^ and 10^−6^ Constrained ML converged better than the EM algorithm, and the two methods performed equally well for 10^−7^ and smaller. The *I*_*F*_ index in Fig. 6 measures the differences between the estimated and true haplotype frequencies, with a value of 1 referring to the scenario when the two are identical. Figure 6 suggests that the two methods behaved similarly for tighter tolerance (10^−6^ and beyond). The *I*_*F*_ for Constrained ML was also more predictable and stable, while there was greater variability for the EM algorithm.

To demonstrate the use of Constrained ML and the relevant LRT we analysed a published dataset on *APOE* [30]. The dataset consists of 9 loci from 80 human individuals whose haplotypes were experimentally identified. We masked the haplotype phase (i.e. as if we obtained their genotypes only) and tried to estimate the haplotype counts for all 36 pairs of loci, and when required, to conduct a LRT to test for the absence of a particular haplotype.

The complete results are presented in Additional file 1, with selected summary in Table 4. For comparison, the results from CubeX and MIDAS (representing the EM algorithm) are also presented [24]. MIDAS was able to correctly estimate the haplotype counts for 28 out of 36 pairs of loci. CubeX provided unique and correct estimates for 26 cases. Additionally in 5 other cases, CubeX provided two solutions, one of which was the correct one. Constrained ML also gave unique and correct haplotype estimates for the same 26 cases as CubeX. LRT were run on the 5 remaining cases that potentially have only 3 haplotypes. LRT made the correct decision on 4 cases (loci pair 1–5, 4–8, 5–7, and 5–9. See Table 4), but falsely rejected the correct answer for loci pair 1–9. To summarise, Constrained ML and LRT jointly provided correct haplotype count estimates for 30 cases.

**Table 4:** Selected results from the analysis of *APOE* dataset

Loci pair	Real count		MIDAS		CubeX 1st solution		CubeX 2nd solution		CML		Possible alternative solution?	CML alternative solution		LRT	CML Decision
1–2	14	116	14	116	14	116	NA		14	116	No	NA		NA	NA
	0	30	0	30	0	30			0	30					
1–3	69	61	64	66	64	66	NA		64	66	No	NA		NA	NA
	17	13	22	8	22	8			22	8					
1–5	120	10	121	9	121	9	120	10	121	9	Yes	120	10	0.15	Accept alternative
	30	0	29	1	29	1	30	0	29	1		30	0		
1–9	9	121	9	121	9	121	12	118	9	121	Yes	12	118	1.84	Accept alternative
	3	27	3	27	3	27	0	30	3	27		0	30		
4–8	98	3	99	2	99	2	98	3	99	2	Yes	98	3	0.06	Accept alternative
	59	0	58	1	58	1	59	0	58	1		59	0		
5–7	141	9	141	9	141	9	131	19	141	9	Yes	131	19	45.91	Reject alternative
	0	10	0	10	0	10	10	0	0	10		10	0		
5–9	12	138	11	139	11	139	12	138	11	139	Yes	12	138	1.37	Accept alternative
	0	10	1	9	1	9	0	10	1	9		0	10		

Selected results from the analysis of *APOE* dataset. The second column shows the real haplotype counts which had been experimentally identified. MIDAS estimates are shown in the next column. CubeX 1st solution refers to the *α* or *β* solution set. CubeX 2nd solution refers to the *γ* solution set should it exist. Constrained ML’s estimates are presented in the sixth column. Log-likelihood was maximised within the entire feasible region. The next step is to decide whether a simpler solution is possible (e.g. there are only 3 haplotypes instead of 4). If we cannot rule of the possibility of having a simpler solution, the log-likelihood is then maximised within the restricted range, with 2 free parameters. LRT statistics are reported, which equal 2 times the differences between the log-likelihoods of the two solutions. If the LRT statistic is greater than ${\chi}_{1,0.95}^2=3.84$, we reject the alternative (simpler) solution at 5% confidence level. Complete results are shown in Additional file 1

## Discussion

### Effect of finite sample size

The theoretical derivation and computer simulations both suggest the observed $E\left[{r}^2\right]=\frac{1}{s}+\left(1-\frac{1}{s}\right){r}_{true}^2$, where *s* = *n* sampled diploid individuals for unphased data, and *s* = 2*n* for haplotypic data. This is different from most existing formulae, which have the form of $E\left[{r}^2\right]={r}_{true}^2+ correction\ factor$ [7, 27]. The explanation is that most previous derivations were based on the null distribution of the *χ*^2^ statistic for association [30], or equivalently assuming ${r}_{true}^2=0$ [29]. These corrections become less reliable when ${r}_{true}^2>0$. For the limiting case of completely linked loci, our sample size correction (Eqs. 4 and 6) guarantees that the implied $\hat{r_{true}^2}$ is also 1, while the existing form over-corrects for sample size [16]. Further, Tables 2 and 3 show that the slope estimates are significantly different from 1, and thus the term (1 − 1/*s*) should be retained. Although the difference can sometimes be small, it is conceptually important that all the squared correlation coefficients must be bounded between 0 and 1. As pointed out by Sved et al. [7], the exact expression for sample size corrections may contain *o*(*s*^−2^) terms, but are shown to be negligible here.

### Empirical variance of *r*^2^

We pointed out earlier that most existing claims about the variance of *r*^2^ are based on ${r}_{true}^2=0$ and do not apply to a wider range of ${r}_{true}^2$ values. The second simulation investigated empirically the variance of the observed ${r}_{phased}^2$ and ${r}_{unphased}^2$ against their expectations and under various sample sizes. The variance plots in Figures 3 and 4 look like parabolas, in which the variances first increase and peak at *E*[*r*^2^] = 0.5 and then come down for larger values. Empirically speaking, the variances go like 2*E*[*r*^2^](1 − *E*[*r*^2^])/*n* for most ${r}_{true}^2>0$ as modelled by the red lines in the plots. It is expected that the two marginal frequencies may play a role in the variance, but the exact expression is too complicated to be evaluated. This approximate formula provides a quick and direct way to approximate the variances and subsequently the confidence intervals of *r*^2^. In addition, this formula helps predict the gain in precision by phasing the data or by increasing the sample size.

### Estimating haplotype frequencies from unphased data

This work proposes a new routine, Constrained ML, to estimate haplotype frequencies from genotypes under HWE. In theory, Constrained ML, EM, and CubeX all aim to maximise the same Hill 1974 log-likelihood function and hence should be identical. In reality they may produce inconsistent results because of the different ways of maximisation. CubeX estimates the haplotype frequencies by solving the cubic equation for the two-locus two-allele case. It may return two sets of answers which are both real and biologically feasible, and this is particularly common when the sample size is small, or when the loci depart from HWE [5]. Another explanation of having multiple answers is that being a root of the cubic equation is only a necessary condition for maximising the likelihood. It is unfortunate that CubeX does not provide any indications on which set of haplotype frequencies we should accept, other than using our “prior knowledge of the LD structure” [5]. For the more general case with multiple alleles, the EM algorithm was introduced because direct maximisation was not always available. It experiences other computing challenges, for example, if *p*_*AB*_*p*_*ab*_ + *p*_*Ab*_*p*_*aB*_ = 0 in any intermediate E-step, the computation halts as division by zero is not permitted. The method is also known to be sensitive to initial conditions, and often to converge to a local rather than the global maximum [21]. With our new method, Constrained ML, the same log-likelihood can be directly maximised within the transformed feasible region. Optimisation within this box-like constraint is a well-studied problem with many routines available across platforms and programming languages, such as L-BFGS-B used in this study. The last simulation compared the convergence between EM and Constrained ML under different sample sizes and stopping criterion. The two methods performed similarly for very tight tolerance for a simple two-allele two-locus setting. A looser relative tolerance is normally implemented in real applications to balance between accuracy and computing time, and in this case Constrained ML produced better convergence than the EM algorithm. Additionally, like the EM algorithm, Constrained ML can handle loci with multiple alleles, by transforming haplotype frequencies into higher-dimensional “cubes” (Additional file 2). The idea of the LRT can also be extended to multiallelic cases to test for the absence of any particular haplotypes. Further comparisons of these methods, especially under more challenging conditions, would be welcome. The *APOE* dataset, with reasonable sample size and often extreme haplotype counts, illustrates the use of Constrained ML and the associated LRT in real applications. The EM-based MIDAS, which provides one estimate a time, got the least correct cases. Although CubeX apparently gave more correct haplotype count estimates, there were 5 ambiguous cases with two solutions. For the 5 loci pairs that potentially have only 3 haplotypes instead of 4, LRT correctly identified the answer in 4 cases, but marginally rejected the correct answer for the loci pair 1–9 at 5% *α* level (LRT statistic = 1.84, *p*-value = 0.17). We should also point out estimation errors were rare but unavoidable, and this is exactly why phased data is preferred. Nonetheless, LRT provides a valuable metric to help decide which set of answer we should accept.

There exist some other methods, such as the Burrows’ method [3, 7], to estimate *r*^2^ without assuming HWE, but they are beyond the scope of this work. Burrows’ Δ measures the so-called composite linkage disequilibrium from non-gametic frequencies, which takes the departure from HWE into account. One can further break down the nine genotypes into eight parameters to include the single-locus disequilibria and higher-order disequilibria [31]. On the downside, they are not as efficient as the likelihood estimators if the HWE assumption is valid.

## Conclusions

This work generalised the estimation of *r*^2^ to all levels of LD, and for both phased and unphased data. New formulae were provided to correct for finite sample size during *r*^2^ point estimation. We approximated the empirical variance of *r*^2^ based on computer simulations. Lastly, a new framework called Constrained ML was suggested to directly estimate haplotype frequencies from diploid genotypic data under HWE. Most inferences utilising LD information will benefit from our new findings.

## Methods

### Computer simulation 1: effect of finite sample size

Simulations were run to verify whether the effect of finite sample size on *r*^2^ estimates is the same as described by Eqs. 3 and 5. First, to ensure most haplotype combinations are covered, a set of true haplotype frequencies was drawn randomly from the uniform *Dirichlet*(1, 1, 1, 1) distribution, which was used to calculate the underlying ${r}_{true}^2$. Second, haplotypes were sampled with a known sample size via the multinomial distribution, and the observed ${r}_{phased}^2$ were calculated via Eq. 1. For unphased case, two haplotypes were paired into one genotype. Haplotype frequencies and ${r}_{unphased}^2$ were estimated through Constrained ML. These two steps were repeated for 10,000 times per sample size, and further repeated for sample sizes of 20, 40, 60, and 80 diploid individuals. The observed ${r}_{phased}^2$ and ${r}_{unphased}^2$ were plotted against ${r}_{true}^2$ for each sample size.

### Computer simulation 2: empirical variance of *r*^2^

Another set of simulations was run to explore the empirical variance of ${r}_{phased}^2$ (or ${r}_{unphased}^2$). The procedure was very similar to the first simulation. For each ${r}_{true}^2$, 500 additional samples were simulated to calculate the variance of the observed ${r}_{phased}^2$ (or ${r}_{unphased}^2$). This was repeated for 10,000 different sets of true haplotype frequencies per sample size, and further repeated for sample sizes of 20, 40, 60, and 80 diploid individuals.

### Computer simulation 3: estimating haplotype frequencies from unphased data

The final set of simulations studied the convergence of Constrained ML and the EM algorithm against different stopping criterion and sample sizes. We measured convergence by two metrics, the relative log-likelihood [32], and the *I*_*F*_ index [21]. For each simulation, true haplotype frequencies were drawn from the *Dirichlet*(1, 1, 1, 1) distribution, which were then used to sample the genotypes with a known sample size. Two haplotypes were randomly paired up to form a genotype. All initially fixed/extinct loci were discarded and resampled. Then the log-likelihood function (Eq. 5) was maximised via Constrained ML and the EM algorithm. In particular, Constrained ML was optimised by the L-BFGS-B routine within the optim() function in R [33, 34]. To avoid false convergence under a specific initial condition, 100 initial conditions were applied to each set of genotypes and the estimate with the largest maximised log-likelihood was used. The whole simulation was repeated 500 times, and further repeated for several different sample sizes and stopping criterion. We used relative tolerance as our stopping criteria, ranging between 10^−2^ and 10^−9^.

## Supplementary information

**Additional file 1. **Complete results from the analysis of *APOE* dataset.

**Additional file 2.** Generalisation of Constrained ML to multiallelic loci.

## Data Availability

The raw *APOE* dataset can be found in the original publication [30]. Computer simulations and mathematical derivations are replicable per instructed in the main text. All computer codes are available upon request. An online program to implement Constrained ML can be found at https://haplotype.shinyapps.io/constrainedml/.

## Abbreviations

CI: Confidence interval
EM: Expectation-Maximisation
HWE: Hardy-Weinberg Equilibrium
LD: Linkage disequilibrium
LRT: Likelihood-ratio test
MLE: Maximum-Likelihood estimator/estimate
