# Supplementary material for: Estimating linkage disequilibrium from genotypes under Hardy-Weinberg equilibrium
Source: BMC Genet. 2020 Feb 26;21:21. doi: 10.1186/s12863-020-0818-9 (PMC7045472; doi:10.1186/s12863-020-0818-9)
Supplement: Supplementary file 1 — Additional file 1. Complete results from the analysis of APOE dataset. [file 12863_2020_818_MOESM1_ESM.docx]

Additional file 1. Complete results from the analysis of *APOE* dataset.

| Loci pair | Real count | | MIDAS | | CubeX 1^st^ solution | | CubeX 2^nd^ solution | | CML | | Possible alternative solution? | CML alternative solution | | LRT | CML Decision |
| --- | --- | --- | --- | --- | --- | --- | --- | --- | --- | --- | --- | --- | --- | --- | --- |
| 1-2 | 14 | 116 | 14 | 116 | 14 | 116 | NA | | 14 | 116 | No | NA | | NA | NA |
|  | 0 | 30 | 0 | 30 | 0 | 30 |  |  | 0 | 30 |  |  |  |  |  |
| 1-3 | 69 | 61 | 64 | 66 | 64 | 66 | NA | | 64 | 66 | No | NA | | NA | NA |
|  | 17 | 13 | 22 | 8 | 22 | 8 |  |  | 22 | 8 |  |  |  |  |  |
| 1-4 | 80 | 50 | 73 | 57 | 73 | 57 | NA | | 73 | 57 | No | NA | | NA | NA |
|  | 21 | 9 | 28 | 2 | 28 | 2 |  |  | 28 | 2 |  |  |  |  |  |
| 1-5 | 120 | 10 | 121 | 9 | 121 | 9 | 120 | 10 | 121 | 9 | Yes | 120 | 10 | 0.15 | Accept alternative |
|  | 30 | 0 | 29 | 1 | 29 | 1 | 30 | 0 | 29 | 1 |  | 30 | 0 |  |  |
| 1-6 | 129 | 1 | 129 | 1 | 129 | 1 | NA | | 129 | 1 | No | NA | | NA | NA |
|  | 30 | 0 | 30 | 0 | 30 | 0 |  |  | 30 | 0 |  |  |  |  |  |
| 1-7 | 117 | 13 | 120 | 10 | 120 | 10 | NA | | 120 | 10 | No | NA | | NA | NA |
|  | 24 | 6 | 21 | 9 | 21 | 9 |  |  | 21 | 9 |  |  |  |  |  |
| 1-8* | 127 | 3 | 127 | 3 | 127 | 3 | 127 | 3 | 127 | 3 | Yes | 127 | 3 | NA | See remarks |
|  | 30 | 0 | 30 | 0 | 30 | 0 | 30 | 0 | 30 | 0 |  | 30 | 0 |  |  |
| 1-9 | 9 | 121 | 9 | 121 | 9 | 121 | 12 | 118 | 9 | 121 | Yes | 12 | 118 | 1.84 | Accept alternative |
|  | 3 | 27 | 3 | 27 | 3 | 27 | 0 | 30 | 3 | 27 |  | 0 | 30 |  |  |
| 2-3 | 9 | 5 | 7 | 7 | 7 | 7 | NA | | 7 | 7 | No | NA | | NA | NA |
|  | 77 | 69 | 79 | 67 | 79 | 67 |  |  | 79 | 67 |  |  |  |  |  |
| 2-4 | 9 | 5 | 7 | 7 | 7 | 7 | NA | | 7 | 7 | No | NA | | NA | NA |
|  | 92 | 54 | 94 | 52 | 94 | 52 |  |  | 94 | 52 |  |  |  |  |  |
| 2-5 | 14 | 0 | 14 | 0 | 14 | 0 | NA | | 14 | 0 | No | NA | | NA | NA |
|  | 136 | 10 | 136 | 10 | 136 | 10 |  |  | 136 | 10 |  |  |  |  |  |
| 2-6 | 14 | 145 | 14 | 145 | 14 | 145 | NA | | 14 | 145 | No | NA | | NA | NA |
|  | 1 | 0 | 1 | 0 | 1 | 0 |  |  | 1 | 0 |  |  |  |  |  |
| 2-7 | 14 | 0 | 14 | 0 | 14 | 0 | NA | | 14 | 0 | No | NA | | NA | NA |
|  | 127 | 19 | 127 | 19 | 127 | 19 |  |  | 127 | 19 |  |  |  |  |  |
| 2-8 | 14 | 143 | 14 | 143 | 14 | 143 | NA | | 14 | 143 | No | NA | | NA | NA |
|  | 3 | 0 | 3 | 0 | 3 | 0 |  |  | 3 | 0 |  |  |  |  |  |
| 2-9 | 9 | 5 | 9 | 5 | 9 | 5 | NA | | 9 | 5 | No | NA | | NA | NA |
|  | 3 | 143 | 3 | 143 | 3 | 143 |  |  | 3 | 143 |  |  |  |  |  |
| 3-4 | 83 | 3 | 83 | 3 | 83 | 3 | NA | | 83 | 3 | No | NA | | NA | NA |
|  | 18 | 56 | 18 | 56 | 18 | 56 |  |  | 18 | 56 |  |  |  |  |  |
| 3-5 | 86 | 0 | 86 | 0 | 86 | 0 | NA | | 86 | 0 | No | NA | | NA | NA |
|  | 64 | 10 | 64 | 10 | 64 | 10 |  |  | 64 | 10 |  |  |  |  |  |
| 3-6 | 86 | 73 | 86 | 73 | 86 | 73 | NA | | 86 | 73 | No | NA | | NA | NA |
|  | 1 | 0 | 1 | 0 | 1 | 0 |  |  | 1 | 0 |  |  |  |  |  |
| 3-7 | 83 | 3 | 83 | 3 | 83 | 3 | NA | | 83 | 3 | No | NA | | NA | NA |
|  | 58 | 16 | 58 | 16 | 58 | 16 |  |  | 58 | 16 |  |  |  |  |  |
| 3-8 | 83 | 3 | 83 | 3 | 83 | 3 | NA | | 83 | 3 | No | NA | | NA | NA |
|  | 74 | 0 | 74 | 0 | 74 | 0 |  |  | 74 | 0 |  |  |  |  |  |
| 3-9 | 12 | 74 | 12 | 74 | 12 | 74 | NA | | 12 | 74 | No | NA | | NA | NA |
|  | 0 | 74 | 0 | 74 | 0 | 74 |  |  | 0 | 74 |  |  |  |  |  |
| 4-5 | 91 | 10 | 91 | 10 | 91 | 10 | NA | | 91 | 10 | No | NA | | NA | NA |
|  | 59 | 0 | 59 | 0 | 59 | 0 |  |  | 59 | 0 |  |  |  |  |  |
| 4-6 | 101 | 58 | 101 | 58 | 101 | 58 | NA | | 101 | 58 | No | NA | | NA | NA |
|  | 1 | 0 | 1 | 0 | 1 | 0 |  |  | 1 | 0 |  |  |  |  |  |
| 4-7 | 82 | 19 | 82 | 19 | 82 | 19 | NA | | 82 | 19 | No | NA | | NA | NA |
|  | 59 | 0 | 59 | 0 | 58 | 0 |  |  | 58 | 0 |  |  |  |  |  |
| 4-8 | 98 | 3 | 99 | 2 | 99 | 2 | 98 | 3 | 99 | 2 | Yes | 98 | 3 | 0.06 | Accept alternative |
|  | 59 | 0 | 58 | 1 | 58 | 1 | 59 | 0 | 58 | 1 |  | 59 | 0 |  |  |
| 4-9 | 12 | 89 | 12 | 89 | 12 | 89 | NA | | 12 | 89 | No | NA | | NA | NA |
|  | 0 | 59 | 0 | 59 | 0 | 59 |  |  | 0 | 59 |  |  |  |  |  |
| 5-6 | 149 | 1 | 149 | 1 | 149 | 1 | NA | | 149 | 1 | No | NA | | NA | NA |
|  | 10 | 0 | 10 | 0 | 10 | 0 |  |  | 10 | 0 |  |  |  |  |  |
| 5-7 | 141 | 9 | 141 | 9 | 141 | 9 | 131 | 19 | 141 | 9 | Yes | 131 | 19 | 45.91 | Reject alternative |
|  | 0 | 10 | 0 | 10 | 0 | 10 | 10 | 0 | 0 | 10 |  | 10 | 0 |  |  |
| 5-8 | 147 | 3 | 147 | 3 | 147 | 3 | NA | | 147 | 3 | No | NA | | NA | NA |
|  | 10 | 0 | 10 | 0 | 10 | 0 |  |  | 10 | 0 |  |  |  |  |  |
| 5-9 | 12 | 138 | 11 | 139 | 11 | 139 | 12 | 138 | 11 | 139 | Yes | 12 | 138 | 1.37 | Accept alternative |
|  | 0 | 10 | 1 | 9 | 1 | 9 | 0 | 10 | 1 | 9 |  | 0 | 10 |  |  |
| 6-7 | 140 | 19 | 140 | 19 | 140 | 19 | NA |  | 140 | 19 | No | NA | | NA | NA |
|  | 1 | 0 | 1 | 0 | 1 | 0 |  |  | 1 | 0 |  |  |  |  |  |
| 6-8 | 156 | 3 | 156 | 3 | 156 | 3 | NA |  | 156 | 3 | No | NA | | NA | NA |
|  | 1 | 0 | 1 | 0 | 1 | 0 |  |  | 1 | 0 |  |  |  |  |  |
| 6-9 | 12 | 147 | 12 | 147 | 12 | 147 | NA |  | 12 | 147 | No | NA | | NA | NA |
|  | 1 | 0 | 1 | 0 | 1 | 0 |  |  | 1 | 0 |  |  |  |  |  |
| 7-8 | 138 | 3 | 138 | 3 | 138 | 3 | NA |  | 138 | 3 | No | NA | | NA | NA |
|  | 19 | 0 | 19 | 0 | 19 | 0 |  |  | 19 | 0 |  |  |  |  |  |
| 7-9* | 12 | 129 | 12 | 129 | 12 | 129 | 12 | 129 | 12 | 129 | Yes | 12 | 129 | NA | See remarks |
|  | 0 | 19 | 0 | 19 | 0 | 19 | 0 | 19 | 0 | 19 |  | 0 | 19 |  |  |
| 8-9 | 12 | 145 | 12 | 145 | 12 | 145 | NA |  | 12 | 145 | No | NA | | NA | NA |
|  | 3 | 0 | 3 | 0 | 3 | 0 |  |  | 3 | 0 |  |  |  |  |  |

Full results from the analysis of *APOE* dataset. The second column shows the real haplotype counts which had been experimentally identified. Haplotype counts were estimated from genotypic data via the three methods: MIDAS, CubeX, and Constrained ML. MIDAS estimates are shown in the next column. CubeX 1^st^ solution refers to the $\alpha$ or $\beta$ solution set. CubeX 2^nd^ solution refers to the $\gamma$ solution set should it exist. Constrained ML’s estimates are presented in the sixth column. Log-likelihood was maximised within the entire feasible region. The next step is to decide whether a simpler solution is possible (e.g. there are only 3 haplotypes instead of 4). If we cannot rule of the possibility of having a simpler solution, the log-likelihood is then maximised within the restricted range, with 2 free parameters. LRT statistics are reported, which equal 2 times the differences between the log-likelihoods of the two solutions. If the LRT statistic is greater than $\chi_{1, 0.95}^{2}=3.84$, we reject the alternative (simpler) solution at 5% confidence level. Complete result table is in supplementary data. Remarks (*): CubeX and Constrained ML provide two sets of haplotype frequency estimates for these cases, but their rounded haplotype counts are the same.
