# Supplementary material for: Estimating linkage disequilibrium from genotypes under Hardy-Weinberg equilibrium
Source: BMC Genet. 2020 Feb 26;21:21. doi: 10.1186/s12863-020-0818-9 (PMC7045472; doi:10.1186/s12863-020-0818-9)
Supplement: Supplementary file 2 — Additional file 2. Generalisation of Constrained ML to multiallelic loci. [file 12863_2020_818_MOESM2_ESM.docx]

Additional file 2. Constrained ML in multiallelic loci.

Constrained ML provides a way to estimate haplotype frequencies from unphased genotypic data through directly maximising the log-likelihood. This Supplementary File explains how this idea can be extended beyond the two biallelic case.

Let $k$ be the number of diploid haplotypes, and $p_{1},p_{2},\ldots, p_{k}$ be the corresponding haplotype frequencies. Similarly, let $m$ be the number of diploid genotypes, and $f_{1},f_{2},\ldots, f_{m}$ be the expected genotype frequencies under HWE. By assuming multinomial sampling, the likelihood of the underlying haplotype frequencies, given the observed counts of each genotypes $n_{1},n_{2},\ldots, n_{m}$ is

$$L\left( p_{1},p_{2},\ldots, p_{k} \right)=\frac{n!}{n_{1}!n_{2}!\ldots n_{m}!}f_{1}^{n_{1}}f_{2}^{n_{2}}\ldots f_{m}^{n_{m}}$$

where $n$ is the total diploid sample size. The expected genotype frequencies $f_{1}, f_{2}, \ldots, f_{m}$ under HWE can be calculated by summing the probabilities of the possible haplotype pairs. Detailed descriptions can be found in Equations 2-3 in Excoffier and Slatkin (cited as [25] in the main text). Each haplotype frequency alone is bounded between 0 and 1, and they sum to one:

$$\sum_{j=1}^{k} p_{j}=1$$

The next step is to transform the haplotype frequencies $p_{1},p_{2},\ldots, p_{k}$ into a $(k-1)$-dimensional “cube”, with new coordinates $u_{1},u_{2},\ldots, u_{k-1}$, via the transformation below:

$$u_{i}=\frac{\sum_{j=1}^{i} p_{j}}{\sum_{j=1}^{i+1} p_{j}}, for i=1, 2, \ldots, k-1$$

The log-likelihood can be maximised by a standard box-like constraint optimisation routine. The backward transformation to obtain the haplotype frequency estimates is:

$$\sum_{j=1}^{i} p_{j}=\prod_{j=i}^{k-1} u_{j}, for i=1, 2, \ldots, k-1$$

$$p_{k}=1-\sum_{j=1}^{k-1} p_{j}$$
